# Feasibility of sentinel lymph node biopsy in breast cancer patients clinically suspected of axillary lymph node metastasis on preoperative imaging

**DOI:** 10.1186/1477-7819-11-104

**Published:** 2013-05-21

**Authors:** Hee Yong Kwak, Byung Joo Chae, Ja Seong Bae, Eun Jin Kim, Eun Young Chang, Sang Hoon Kim, Sang Seol Jung, Byung Joo Song

**Affiliations:** 1 Department of Surgery, Catholic University of Korea, College of Medicine, Seoul, Republic of Korea

**Keywords:** Breast, Lymph node, Metastasis, Sentinel lymph node biopsy

## Abstract

**Background:**

Generally, sentinel lymph node biopsy (SLNB) is performed in patients with clinically negative axillary lymph node (LN). This study was to assess imaging techniques in axillary LN staging and to evaluate the feasibility of SLNB in patients clinically suspected of axillary LN metastasis on preoperative imaging techniques (SI).

**Methods:**

A prospectively maintained database of 767 breast cancer patients enrolled between January 2006 and December 2009 was reviewed. All patients were offered preoperative breast ultrasound, magnetic resonance imaging, and positron emission tomography scanning. SI patients were regarded as those for whom preoperative imaging was “suspicious for axillary LN metastasis” and NSI as “non-suspicious for axillary LN metastasis” on preoperative imaging techniques. Patients were subgrouped by presence of SI and types of axillary operation, and analyzed.

**Results:**

For 323 patients who received SLNB, there was no statistically significant difference in axillary recurrence (*P*=0.119) between SI and NSI groups. There also was no significant difference in axillary recurrence between SLNB and axillary lymph node dissection (ALND) groups in 356 SI patients (*P*=0.420). The presence of axillary LN metastasis on preoperative imaging carried 82.1% sensitivity and 45.9% specificity for determining axillary LN metastasis on the final pathology.

**Conclusions:**

SLNB in SI patents is safe and feasible. Complications might be avoided by not performing ALND. Therefore, we recommend SLNB, instead of a direct ALND, even in SI patients, for interpreting the exact nodal status and avoiding unnecessary morbidity by performing ALND.

## Background

Sentinel lymph node biopsy (SLNB) is the preferred method to assess the pathologic status of the axillary lymph nodes (LNs) for patients with stage I or II breast cancer [1-8]. SLNB has been recommended based on the results of recent randomized clinical trials showing decreased arm and shoulder morbidity in patients with breast cancer undergoing SLNB compared with those undergoing standard axillary lymph node dissection (ALND) [5,9]. However, not all patients are candidates for SLNB.

According to the National Comprehensive Cancer Network (NCCN) guideline, SLNB should be performed in patients with clinically negative axillary LNs [10]. Yet, SLNB is still not recommended by the American Society of Clinical Oncology (ASCO) for large or locally advanced invasive breast cancers (T3 and T4), inflammatory breast cancer, during pregnancy, in the setting of prior non-oncologic breast surgery or axillary surgery, and in the presence of suspicious palpable axillary LN. In the ASCO guideline recommendations, the sensitivity of SLN biopsy for node involvement ranged from 71% to 100%, and the false negative rate averaged 8.5% across 69 studies (10,454 patients) analyzed [1]. Nevertheless, the role of SLNB is increasing and even palpable axillary LN alone is no longer a contraindication [11].

In the era of SLNB, the exclusion of LN metastases using noninvasive methods could reduce the rate of axillary surgery [12]. However, the experience of the examiner is important for the diagnostic precision and prediction. Therefore, the accuracy of the imaging study has appropriately been questioned. It is well known that sensitivity and positive predictive value of preoperative axillary ultrasound (US) are low [13] and those of the positron emission tomography (PET) scan are even lower [14].

The aim of this study was to assess the imaging techniques in axillary LN staging and to evaluate the feasibility of SLNB even in patients clinically suspected of axillary LN metastasis on preoperative imaging techniques (SI).

## Methods

### Patient cohort

Between January 2006 and December 2009, 767 consecutive patients with biopsy-proven invasive breast cancer were successfully treated by two breast surgeons. All the patients were offered preoperative imaging techniques including breast US, contrast-enhanced magnetic resonance imaging (MRI), and PET scanning. Preoperative MRI was done to determine the extent of surgery (for example, wide excision or simple mastectomy). PET-CT was performed to find out whether any distant metastasis existed before the operation. Along with mammography and ultrasound, these are routine preoperative examinations in our hospital. All clinical and pathologic features were collected prospectively from the database of Seoul St Mary’s Hospital.

Exclusion criteria were male gender, previous breast cancer surgery, prior breast irradiation, known distant metastasis, prior axillary surgery, inflammatory breast cancer, and neoadjuvant chemotherapy. In the remaining 571 patients, SLN biopsy or ALND was performed. Before May 2008, patients with clinically suspicious axillary LN were treated with ALND. Consequently, 323 patients underwent SLNB and the remainder (n=248) ALND. All protocols were approved by the institutional review committee of the Seoul St Mary’s Hospital (KC12RISI0396) and met the guidelines of the responsible governmental agency.

### Detection and false negative rate

SI patients were regarded as those for whom any of preoperative imaging techniques (that is, US, MRI, and PET scan) proved suspicious for axillary LN metastasis. The detection rate was determined based on patients who underwent successful SLNB and did not experience conversion to ALND. Unscheduled ALND due to unsuccessful SLNB was considered a failure and was used to determine the failure rate. Axillary recurrence after surgery represented a false negative and was used to determine the false negative rate.

### Imaging protocols

Axillary US was done by two experienced breast radiologists using a Seimens SEQUOIA 512 (Acuson, Mountain View, CA, USA) equipped with a broad-band linear probe (5 to 12 MHz). As in a recent study [15], axillary LN was classified on the basis of cortical thickness (cutoff 2.5 mm) and the appearance (for example, an irregular nodular cortex, a diminished or absent fatty hilum, or cortical thickening greater than 3 mm) on ultrasound for predicting the presence of axillary metastasis. MRI was performed with a 1.5 tesla signa (GE Medical Systems, Milwaukee, WI, USA) with fat-suppressed fast spin-echo T2-weighted imaging (TR/TE=4000/85, flip angle 90 degrees, 30 slices with field of view (FOV) of 240 mm, matrix 256 × 224, two NEX and 3 mm section thickness with 0.1 mm intersection gap, acquisition time 2 minutes, 56 sec) and interpreted by two breast radiologists. PET-CT scanning (Biography LSO; Siemens Medical Solutions, Knoxville, TN, USA) was performed after 8 hours of fasting. Patients were injected intravenously with 12.5 mCi of 2-(fluorine-18)-fluoro-deoxy-D-glucose (F-18 FDG) in the contralateral hand. One hour after injection, patients were positioned in the scanner with their arms above their heads. Attenuation correction of images was not performed. A single nuclear physician who was aware of the diagnosis of breast carcinoma interpreted the scans.

### Sentinel lymph node biopsy

Before 2010, SLNB was performed using the blue dye method in our hospital because we had no isotope or gamma probe before the year 2010; we used a standard 1-day protocol using the blue dye for localization. Briefly, at the operation, 4 ml of 0.8% Indigocarmine Blue dye (Carmine; Korea United Pharm., Seomyoen, Chungnam, South Korea) was used. Injections were given subdermally into the upper and outer areolar borders. If a node was blue or suspicious, it was considered a sentinel lymph node (SLN). A 5-cm incision along the anteroinferior border or the axillary hairline was done and SLNs were retrieved manually and sent for frozen section analysis.

### Histologic evaluation

SLNs or nodes were sliced at approximately 2-mm intervals along the longitudinal axis of the LN. The largest slice from each SLN was subjected to immediate frozen-section examination with hematoxylin and eosin (H&E) staining and standard evaluation was assessed for the presence of metastases by an experienced breast pathologist. If a frozen section confirmed the presence of metastases in the SLN, completion ALND was done. The remaining tissue of the axillary SLNs was fixed in formalin and embedded in paraffin for the permanent pathologic report. The pathologist assessed lymph nodes with H&E staining only. When the final diagnosis of axillary LN metastasis was made, we performed additional ALND. All of patients with axillary LN metastasis underwent adjuvant chemotherapy.

### Estrogen receptor, progesterone receptor, and HER2 status

Estrogen receptor (ER), progesterone receptor (PR) and HER2 status was reviewed from medical records. Hormone receptor status was determined using an enzyme immunoassay and reported in the medical records between 2006 and 2009. The receptor status had been determined using a commercial enzyme immunoassay according to the instructions of the manufacturer (Abbott Laboratories, Chicago, IL, USA). A result exceeding 15 fmol/mg was considered positive for the presence of the particular receptor. Tissue microarray (TMA) of primary breast tissue was used for analysis of HER2 overexpression. Immunohistochemistry (IHC) or fluorescence *in situ* hybridization (FISH) for evaluating HER2 status was performed, and an IHC score of three positive or FISH-positive was defined as positive for HER2 overexpression. The IHC method was briefly as follows; five-micrometer sections of paraffin-embedded tissue arrays were deparaffinized, rehydrated in a graded series of alcohol solutions and microwave-treated for 10 minutes in a pH 6.0 citrate buffer. The endogenous peroxidase activity was blocked using 0.3% hydrogen peroxide. The tissue arrays were processed in an automatic IHC staining machine using standard procedures (Lab Vision autostainer; Lab Vision, Fremont, CA, USA) and a ChemMate™ EnVision™ system (DAKO, Carpinteria, CA, USA). FISH was performed using the PathVysion™ HER2/CEN probe (Vysis, Downers Grove, IL, USA). The c-erbB2 to chromosome 17 centromere ratio was measured in at least 60 nuclei from the tumor cells, and an average score was taken. More than two copies of c-erbB2 for each chromosome 17 were considered to be a positive sign for c-erbB2 gene amplification.

### Statistical analyses

The primary endpoint was axillary recurrence. Adjuvant therapy comprising chemotherapy and/or radiotherapy was performed for all the patients who underwent an operation according to the standard protocols used in our hospital. Axillary recurrence-free survival (ARFS) was defined as the time from definitive breast surgery to first documented axillary recurrence. The results were presented as the mean ± SD or number (%) as appropriate. The chi-square test was used to compare categorical variables, and the independent two-sample *t*-test was used to compare the values of continuous variables between groups. The Kaplan-Meier method was used for survival analysis and the log rank test for estimating the axillary ARFS. All statistical tests were two-sided, and a *P*-value < 0.05 was considered statistically significant.

## Results

One hundred ninety six patients were excluded, leaving 571 patients for the study. Median follow up was 45 months (range, 1 to 73 months). Factors evaluated for clinical and pathologic features were mean age of the patients, axillary recurrence, recurrence, tumor (T) and node (N) stage, pathologic stage, size of tumor, nuclear and histologic grade, ER status, PR status, IHC, and lymphovascular invasion. Axillary recurrence occurred in 6 of 323 patients in the SLNB group, and 10 of 248 patients in the ALND group (Table 1).

**Table 1:** Clinical and pathologic characteristics of 571 patients

**Characteristics**	**SLNB(n=323)**	**ALND(n=248)**	***P*(Chi-square test)**
Mean age ±SD	51.06 ± 9.80	51.38 ± 9.54	0.540
Axillary recurrence			0.119
No	317	238	
Yes	6	10	
Recurrence			0.001
No	302	209	
Yes	20	39	
T stage			0.001
pT1	232	127	
pT2	79	104	
pT3	12	15	
pT4	0	2	
N stage			0.001
pN0	256	147	
pN1	52	85	
pN2	8	6	
pN3	7	10	
Stage			0.001
Stage I	199	87	
Stage IIA	78	94	
Stage IIB	29	40	
Stage IIIA	10	16	
Stage IIIB	0	2	
Stage IIIC	7	9	
Size of tumor, cm, mean ± SD	1.84 ± 1.55	2.56 ± 2.04	0.001
Histology			0.003
G1	100	47	
G2	147	73	
G3	62	43	
Nuclear			0.015
G1	50	32	
G2	203	136	
G3	63	74	
ER			0.495
Negative	96	80	
Positive	227	167	
PR			0.311
Negative	119	102	
Positive	203	146	
IHC			0.722
Negative/1+	175	131	
2+	78	66	
3+	69	48	
Lymphovascular invasion			0.001
No	226	137	
Yes	97	111	

SLNB, sentinel lymph node biopsy; ALND; axillary lymph node dissection; ER,estrogen receptor; PR,progesterone receptor; IHC,immunohistochemistry.

Clinical and pathologic features for the 323 patients of the SLNB group were studied and the results were compared with those of patients who were clinically non-suspicious for axillary LN metastasis (NSI) and SI. The results are summarized in Table 2. Mean SLNB and SI patient age was 51.90 and 50.35 years, respectively, and was not significantly different between the two groups (*P*=0.790). Axillary recurrence was noted more frequently in the SI group but not with statistical significance (1/147 vs 5/171, *P*=0.152). The sole factor associated with SI patients was recurrence. Other factors associated with clinical suspicion of lymph node metastasis on univariate analysis were T and N stage, pathologic stage, ER status, nuclear and histologic grade, and lymphovascular invasion.

**Table 2:** Clinical and pathologic characteristics of SLNB for subgroups based on clinical suspicion of lymph node metastasis

**Characteristics**	NSI^*^ (n=147)	SI^*^ (n= 176)	***P*(Chi-square test)**
Mean age ± SD	51.90 ± 9.99	50.35 ± 9.60	0.790
Axillary recurrence			0.152
No	146	171	
Yes	1	5	
Recurrence			0.017
No	143	159	
Yes	4	16	
T stage			0.022
pT1	112	120	
pT2	34	45	
pT3	1	11	
N stage			0.002
pN0	129	127	
pN1	17	35	
pN2	1	7	
pN3	0	7	
Stage			0.002
Stage I	101	98	
Stage IIA	37	41	
Stage IIB	8	21	
Stage IIIA	1	9	
Stage IIIC	0	7	
Size of tumor, cm, mean ± SD	1.58 ± 0.89	2.06 ± 1.91	0.463
Histology			0.013
G1	53	47	
G2	74	73	
G3	19	43	
Nuclear			0.005
G1	28	22	
G2	100	103	
G3	18	45	
ER			0.009
Negative	33	63	
Positive	114	113	
PR			0.167
Negative	48	71	
Positive	98	105	
IHC			0.086
Negative/1+	81	92	
2+	40	38	
3+	23	45	
Lymphovascular invasion			0.013
No	113	113	
Yes	34	63	

^*^Clinically suspicious for axillary LN metastasis on preoperative imaging techniques (SI) patients are those with any of preoperative imaging techniques, including US, MRI, and PET scan, who were suspected of axillary LN metastasis, and NSI are those not suspected of LN metastasis.ER,estrogen receptor; PR,progesterone receptor; IHC,immunohistochemistry.

**Figure 1:**
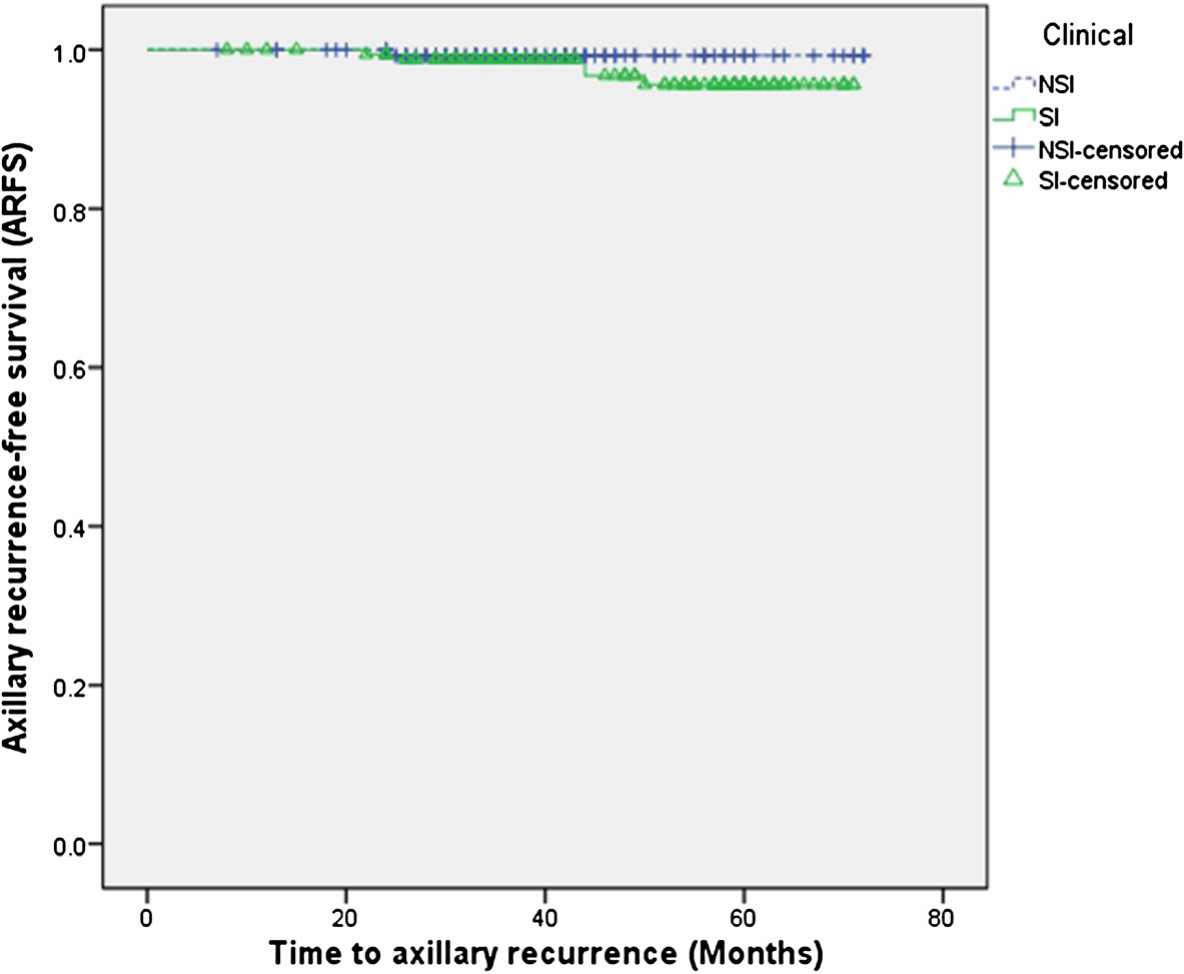
**Evaluation of axillary recurrence-free survival (ARFS) for subgroups based on imaging studies.** SI, clinically suspicious axillary lymph node recurrence on preoperative imaging studies; NSI, clinically non-suspicious axillary lymph node recurrence on preoperative imaging studies.

During the study period, five patients of the SLNB group experienced failure because no sentinel nodes were found during the operation. Therefore, the detection rate of SLN was 98.5% (318/323). The 5-year axillary recurrence rate was 0.7% (1/147) for NSI patients and 2.8% (5/176) for SI patients; the rates were not statistically significant (*P*=0.295) Therefore, ARFS was estimated as 97.2% (171/176) for SI and 99.3% (146/147), respectively (Figure 1) and there was no statistically significant difference in the false negative rate.

**Figure 2:**
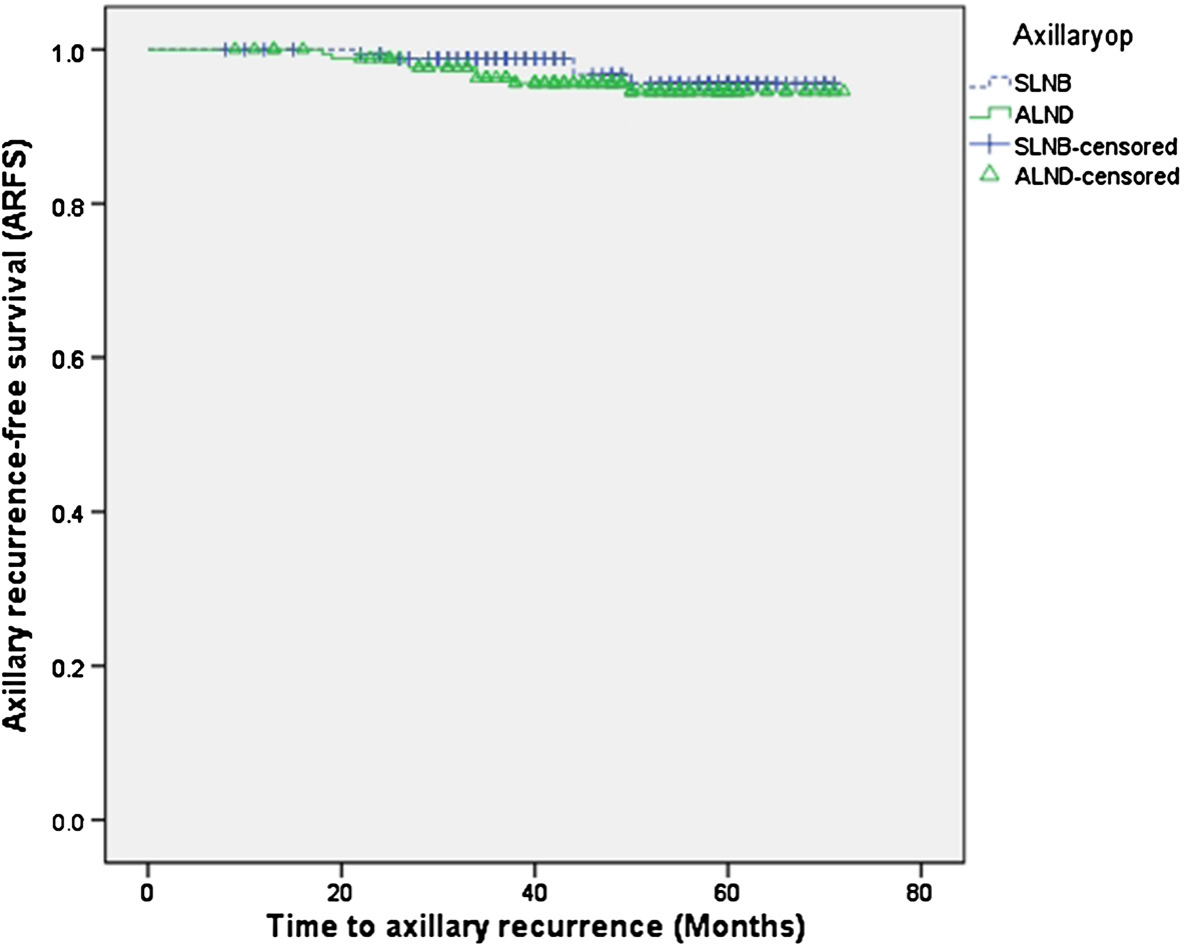
**Evaluation of axillary recurrence-free survival (ARFS) for subgroups based on type of axillary operation.** SLNB, sentinel lymph node biopsy; ALND, axillary lymph node dissection.

Further statistical analyses were done for patients clinically suspected of LN metastasis. The patients were subgrouped as SLNB and ALND (Table 3). There was no statistically significant difference in axillary recurrence between the two groups (5/176 vs 8/180, *P*=0.420) and a Kaplan-Meier curve showed no difference (*P*=0.486, Figure 2).

**Table 3:** Clinical and pathologic characteristics of patients clinically suspected of lymph node metastasis for subgroups based on type of axillary operation

**Characteristics**	**SLNB (n=176)**	**ALND (n= 180)**	***P*(Chi-square test)**
Mean age ± SD	52.00 ± 9.61	47.00 ± 9.45	0.743
Axillary recurrence			0.420
No	171	172	
Yes	5	8	
Recurrence			0.049
No	159	151	
Yes	16	29	
T stage			0.001
pT1	120	86	
pT2	45	78	
pT3	11	14	
pT4	0	2	
N stage			0.001
pN0	127	91	
pN1	35	74	
pN2	7	6	
pN3	7	9	
Stage			0.001
Stage I	98	54	
Stage IIA	41	63	
Stage IIB	21	38	
Stage IIIA	9	15	
Stage IIIB	0	2	
Stage IIIC	7	8	
Size of tumor, cm, mean ± SD	2.06 ± 1.91	2.62 ± 1.70	0.019
Histology			0.013
G1	47	27	
G2	73	96	
G3	43	48	
Nuclear			0.104
G1	22	20	
G2	103	90	
G3	45	65	
ER			0.833
Negative	63	66	
Positive	113	113	
PR			0.311
Negative	71	79	
Positive	105	101	
IHC			0.525
Negative/1+	92	89	
2+	38	47	
3+	46	41	
Lymphovascular invasion			0.002
No	113	63	
Yes	86	94	

ER,estrogen receptor; PR,progesterone receptor; IHC,immunohistochemistry.

The presence of axillary LN metastasis on preoperative imaging carried an 82.1% sensitivity, 45.9% specificity, 38.8% positive predictive value (PPV), and 86.1% negative predictive value (NPV) for determining axillary LN metastasis on final pathology. PET-CT was the most sensitive imaging technique (62.41%) for identifying axillary LN metastasis, followed by MRI (61.73%) and US (59.88%) (Table 4). Specificity for the imaging techniques was 82.96% for PET-CT, 82.09% for US, and 78.24% for MRI. The specificity of SI was low (45.9%) and unnecessary ALND was performed in more than half of the patients.

**Table 4:** Diagnostic accuracy of imaging techniques in staging axillary nodes

	**Sensitivity (%)**	**Specificity (%)**	**PPV (%)**	**NPV (%)**	**Accuracy (%)**
US	59.88	82.09	58.14	83.12	75.57
MRI	61.73	78.24	54.35	82.97	73.36
PET-CT	62.41	82.96	65.67	80.87	75.91
SI^*^	82.14	45.91	38.76	86.05	56.57

^*^SI are patients in whom a preoperative imaging technique, including breast ultrasound (US), contrast-enhanced magnetic resonance imaging (MRI), and positron emission tomography (PET)-CT scanning, revealed suspected axillary LN metastasis. PPV, positive predictive value; NPV, negative predictive value.

## Discussion

The aim of this study was to determine whether it is safe and feasible to perform SLNB in patients with clinically suspicious axillary LN metastasis on preoperative imaging studies. The disease status of the axillary LN is the most significant prognostic factor for patients with breast cancer [16]. SLNB is feasible and accurate, works well in a wide range of practice settings, increases staging accuracy by allowing enhanced pathologic analysis, has less morbidity than ALND, and gives local control comparable to that of ALND [17]. Therefore, ALND for uninvolved axillary LN is considered unnecessary and improper, and indication of SLNB is important. Presently, there was no statistically significant difference in axillary recurrence between SLNB and ALND groups for clinically suspicious axillary LN metastasis (*P*=0.420). Therefore, SLNB in clinically suspicious patients on preoperative imaging studies might be safe and feasible in the clinical setting.

The NCCN guideline recommends that SLNB should be performed in patients with clinically negative axillary LNs [10]. Still, many authorities consider clinically positive axillary LNs a contraindication for SLNB [1]. Nevertheless, the role of SLNB is increasing and various studies have shown that clinical assessment of axillary LNs alone is inaccurate with a false positive rate up to 40% [11,18]. In our study, SI patients were regarded as those for whom the preoperative imaging techniques of US, MRI, and PET-CT scanning revealed suspicion of axillary LN. Owing to the low specificity of imaging studies, direct ALND in SI patients could potentially lead to unnecessary ALND.

This study emphasizes the feasibility of SLNB, even in SI patients. More than 60% of all primary operable breast cancers do not have axillary LN metastasis [12]. Non-invasive methods like US, MRI, PET-CT have gained more importance in staging axillary LN. Nevertheless, no imaging study is completely accurate. Axillary US provides additional value in detecting pathological axillary LN [19,20] but sensitivity has reportedly varied from 26.4% (15.3% to 40.3%) to 75.9% (56.4% to 89.7%) and specificity from 88.4% (82.1% to 93.1%) to 98.1% (90.1% to 99.9%) [21]. A meta-analysis of 25 studies including 2,460 patients reported that PET-CT provided lower sensitivity (37% to 85%) and high specificity (84% to 100%) [22]. The present analysis also indicated that the sensitivity of PET-CT is not sufficient for staging axillary LNs. Also, adding axillary MRI sequentially after axillary US does not significantly improve detection rate of positive nodes [23]. In our study, 56.57% of accuracy (Table 4) was shown with preoperative imaging techniques and false positivity of SI reached 61.23% (218/356) (Table 3). Hence, SLNB in SI patients might be possible.

It is now regarded that a proportion of patients can be spared an SLNB with the aid of preoperative axillary US combined with fine-needle aspiration cytology (FNAC) [24]. Indeed, when FNAC is combined, the likelihood of false positives and false negatives would decrease [13]. However, preoperative axillary US alone is insufficiently specific to obviate the need for SLNB because of the substantial number of false negative results, especially in stage N1 disease, although it may almost exclude N2 and N3 disease [25]. In our study, 52/323 patients (16.1%) who underwent SLNB were proved to have N1 disease. Therefore, preoperative axillary US combined with FNAC alone might have produced false negative results with additional costs. In fact, we tried to perform routine axillary US combined with core needle biopsy but a number of patients (the proportion is not identifiable) refused to perform additional biopsy due to additional pain, cost, and bleeding.

There are some limitations to our study. First, it is a retrospective review of a small number of patients. Further evaluation with a large number of prospective randomized controlled studies must be done in a more standardized group for validation. Second, there were some confounding factors concerning postoperative adjuvant therapies, including radiation, systemic, or endocrine therapies. These factors could have influenced the results. Despite these limitations, the study is significant and is the first to recommend SLNB in SI, based on its demonstrated feasibility and safety.

Our evaluation of SLNB in SI patients with breast cancer suggests that no imaging techniques can replace surgical staging and histologic confirmation of nodal status [14]. Hence the inaccuracy of the imaging techniques allows indications of SLNB widening and SLNB in SI patients is safe and feasible. However, further prospective trials with a larger cohort of patients with long-term follow up are required to verify this observation.

## Conclusions

The inaccuracy of the imaging techniques allows widening of indications of SLNB. Therefore, we recommend SLNB, instead of a direct ALND, even in SI patients, for interpreting the exact nodal status and avoiding unnecessary morbidity by performing ALND.

## Competing interests

The authors declare that they have no competing interests.

## Authors’ contributions

HYK contributed mainly in the design, literature review, and writing of the article. The data were collected and assembled by HYK, EJK, EYC, and SHK. BJC, JSB, and SSJ gave valuable advice and edited the discussion. Both SSJ and BJS provided administrative support. BJS has given final approval for the version to be published. All authors read and approved the final manuscript.

## References

[B1] Lyman GH Giuliano AE Somerfield MR Benson AB 3rd Bodurka DC Burstein HJ Cochran AJ Cody HS 3rd Edge SB Galper S Hayman JA Kim TY Perkins CL Podoloff DA Sivasubramaniam VH Turner RR Wahl R Weaver DL Wolff AC Winer EP American Society of Clinical Oncology American Society of Clinical Oncology guideline recommendations for sentinel lymph node biopsy in early-stage breast cancer J Clin Oncol 2005 11 7703 7720 10.1200/JCO.2005.08.001 16157938

[B2] Cox CE Nguyen K Gray RJ Salud C Ku NN Dupont E Hutson L Peltz E Whitehead G Reintgen D Cantor A Importance of lymphatic mapping in ductal carcinoma in situ (DCIS): why map DCIS? Am Surg 2001 11 513 519 discussion 519–521 11409797

[B3] Bass SS Lyman GH McCann CR Ku NN Berman C Durand K Bolano M Cox S Salud C Reintgen DS Cox CE Lymphatic Mapping and Sentinel Lymph Node Biopsy Breast J 1999 11 288 295 10.1046/j.1524-4741.1999.00001.x 11348304

[B4] Cox CE Lymphatic mapping in breast cancer: combination technique Ann Surg Oncol 2001 11 67S 70S 11599905

[B5] Veronesi U Paganelli G Viale G Luini A Zurrida S Galimberti V Intra M Veronesi P Robertson C Maisonneuve P Renne G De Cicco C Gennari R A randomized comparison of sentinel-node biopsy with routine axillary dissection in breast cancer N Engl J Med 2003 11 546 553 10.1056/NEJMoa012782 12904519

[B6] O’Hea BJ Hill AD El-Shirbiny AM Yeh SD Rosen PP Coit DG Borgen PI Cody HS 3rd Sentinel lymph node biopsy in breast cancer: initial experience at Memorial Sloan-Kettering Cancer Center J Am Coll Surg 1998 11 423 427 10.1016/S1072-7515(98)00060-X 9544956

[B7] Krag D Weaver D Ashikaga T Moffat F Klimberg VS Shriver C Feldman S Kusminsky R Gadd M Kuhn J Harlow S Beitsch P The sentinel node in breast cancer–a multicenter validation study N Engl J Med 1998 11 941 946 10.1056/NEJM199810013391401 9753708

[B8] McMasters KM Giuliano AE Ross MI Reintgen DS Hunt KK Byrd DR Klimberg VS Whitworth PW Tafra LC Edwards MJ Sentinel-lymph-node biopsy for breast cancer–not yet the standard of care N Engl J Med 1998 11 990 995 10.1056/NEJM199810013391410 9753717

[B9] Mansel RE Fallowfield L Kissin M Goyal A Newcombe RG Dixon JM Yiangou C Horgan K Bundred N Monypenny I England D Sibbering M Abdullah TI Barr L Chetty U Sinnett DH Fleissig A Clarke D Ell PJ Randomized multicenter trial of sentinel node biopsy versus standard axillary treatment in operable breast cancer: the ALMANAC Trial J Natl Cancer Inst 2006 11 599 609 10.1093/jnci/djj158 16670385

[B10] Carlson RW Allred DC Anderson BO Burstein HJ Carter WB Edge SB Erban JK Farrar WB Goldstein LJ Gradishar WJ Hayes DF Hudis CA Jahanzeb M Kiel K Ljung BM Marcom PK Mayer IA McCormick B Nabell LM Pierce LJ Reed EC Smith ML Somlo G Theriault RL Topham NS Ward JH Winer EP Wolff AC NCCN Breast Cancer Clinical Practice Guidelines Panel Breast cancer. Clinical practice guidelines in oncology J Natl Compr Canc Netw 2009 11 122 192 19200416 10.6004/jnccn.2009.0012

[B11] Specht MC Fey JV Borgen PI Cody HS 3rd Is the clinically positive axilla in breast cancer really a contraindication to sentinel lymph node biopsy? J Am Coll Surg 2005 11 10 14 10.1016/j.jamcollsurg.2004.09.010 15631914

[B12] Gerber B Heintze K Stubert J Dieterich M Hartmann S Stachs A Reimer T Axillary lymph node dissection in early-stage invasive breast cancer: is it still standard today? Breast Cancer Res Treat 2011 11 613 624 10.1007/s10549-011-1532-0 21523451

[B13] Rattay T Muttalib M Khalifa E Duncan A Parker SJ Clinical utility of routine pre-operative axillary ultrasound and fine needle aspiration cytology in patient selection for sentinel lymph node biopsy Breast 2012 11 210 214 10.1016/j.breast.2011.09.014 21981897

[B14] Lovrics PJ Chen V Coates G Cornacchi SD Goldsmith CH Law C Levine MN Sanders K Tandan VR A prospective evaluation of positron emission tomography scanning, sentinel lymph node biopsy, and standard axillary dissection for axillary staging in patients with early stage breast cancer Ann Surg Oncol 2004 11 846 853 10.1245/ASO.2004.11.033 15313737

[B15] Cho N Moon WK Han W Park IA Cho J Noh DY Preoperative sonographic classification of axillary lymph nodes in patients with breast cancer: node-to-node correlation with surgical histology and sentinel node biopsy results AJR Am J Roentgenol 2009 11 1731 1737 10.2214/AJR.09.3122 19933672

[B16] Goldhirsch A Glick JH Gelber RD Coates AS Thurlimann B Senn HJ Meeting highlights: international expert consensus on the primary therapy of early breast cancer Ann Oncol 2005 11 1569 1583 10.1093/annonc/mdi326 16148022

[B17] Cody HS 3rd Sentinel lymph node biopsy for breast cancer: does anybody not need one? Ann Surg Oncol 2003 11 1131 1132 10.1245/ASO.2003.10.905 14654465

[B18] Lanng C Hoffmann J Galatius H Engel U Assessment of clinical palpation of the axilla as a criterion for performing the sentinel node procedure in breast cancer Eur J Surg Oncol 2007 11 281 284 10.1016/j.ejso.2006.09.032 17084579

[B19] Zgajnar J Hocevar M Podkrajsek M Hertl K Frkovic-Grazio S Vidmar G Besic N Patients with preoperatively ultrasonically uninvolved axillary lymph nodes: a distinct subgroup of early breast cancer patients Breast Cancer Res Treat 2006 11 293 299 10.1007/s10549-005-9123-6 16333526

[B20] Mathijssen IM Strijdhorst H Kiestra SK Wereldsma JC Added value of ultrasound in screening the clinically negative axilla in breast cancer J Surg Oncol 2006 11 364 367 10.1002/jso.20590 16967456

[B21] Alvarez S Anorbe E Alcorta P Lopez F Alonso I Cortes J Role of sonography in the diagnosis of axillary lymph node metastases in breast cancer: a systematic review AJR Am J Roentgenol 2006 11 1342 1348 10.2214/AJR.05.0936 16632729

[B22] Peare R Staff RT Heys SD The use of FDG-PET in assessing axillary lymph node status in breast cancer: a systematic review and meta-analysis of the literature Breast Cancer Res Treat 2010 11 281 290 10.1007/s10549-010-0771-9 20140703

[B23] Garcia Fernandez A Fraile M Gimenez N Rene A Torras M Canales L Torres J Barco I Gonzalez S Veloso E González C Cirera L Pessarrodona A Use of axillary ultrasound, ultrasound-fine needle aspiration biopsy and magnetic resonance imaging in the preoperative triage of breast cancer patients considered for sentinel node biopsy Ultrasound Med Biol 2011 11 16 22 10.1016/j.ultrasmedbio.2010.10.011 21144955

[B24] Cools-Lartigue J Meterissian S Accuracy of axillary ultrasound in the diagnosis of nodal metastasis in invasive breast cancer: a review World J Surg 2012 11 46 54 10.1007/s00268-011-1319-9 22037691

[B25] Choi JS Kim MJ Moon HJ Kim EK Yoon JH False negative results of preoperative axillary ultrasound in patients with invasive breast cancer: correlations with clinicopathologic findings Ultrasound Med Biol 2012 11 1881 1886 10.1016/j.ultrasmedbio.2012.07.011 22975037

